# “The team needs to feel cared for”: staff perceptions of compassionate care, aids and barriers in adolescent mental health wards

**DOI:** 10.1186/s12912-022-00994-z

**Published:** 2022-08-01

**Authors:** Lucy Maddox, Manuela Barreto

**Affiliations:** 1 Department of Psychology, University of Bath, 10 West, Claverton Down, Bath, BA2 7AY UK; 2 Department of Psychology, University of Exeter, Washington Singer Building, Perry Road, Exeter, EX4 4QG UK

**Keywords:** Adolescent mental health staff, Compassion, Compassion fatigue, Mental health, Patient care, Staff wellbeing

## Abstract

**Background:**

Compassion is vital in healthcare. Current understandings of the nature of compassionate care, its aids and barriers, are more theoretically developed than grounded in staff experience. This study explores staff perceptions of compassionate care in child and adolescent mental health wards.

**Methods:**

Three focus groups were conducted with a total of 35 staff from adolescent mental health wards (10–12 people in each group), on the nature of compassionate care, aids and barriers. Transcripts were analysed using thematic analysis. A follow-up survey with 36 workers from other UK child and adolescent mental health wards was completed and means and standard deviations of responses were analysed to confirm wider resonance of themes.

**Results:**

Elements of compassionate care fell into six themes relating to individual, team and organisational factors: emotional connection, sense of being valued, attention to the whole person, understanding, good communication, and practical help/resources. Aids and barriers mirrored each other, and showed that what staff think is key to the nature of compassionate care for patients is also what they feel they need to receive to be able to show compassionate care.

**Conclusions:**

This study suggests that staff need the same elements of compassion as those which they seek to provide. A greater emphasis needs to be placed on providing staff with individual, team and organisational level resources which help them to feel compassionately held within the interconnected systems in which they work, in order to be able to continue to provide high level compassionate care. Staff need to be nourished, valued and compassionately cared for in order to be able to care compassionately for the patients they look after.

**Supplementary Information:**

The online version contains supplementary material available at 10.1186/s12912-022-00994-z.

## Background

A broad definition of compassion is “the feeling or emotion, when a person is moved by the suffering or distress of another, and by the desire to relieve it” [1]. Compassion is vital to healthcare provision since identifying a problem, caring about the outcome, and taking action to solve the problem are key parts of helping an individual recover from illness and/or distress. Compassion is recognised as an important aspect of healthcare in many countries worldwide [2–4] including in the UK’s National Health Service (NHS) Constitution [5], the Dutch-based Charter for Compassion [2] and US-based healthcare providers [6]. Compassionate care has been associated with improved clinical outcomes and improved patient experience [7–10].

The importance of guarding compassionate attitudes in healthcare staff was highlighted in 2013 by the Francis Report, in the UK, which reported serious failings in care in the mid-Staffordshire healthcare trust and emphasized the devastating, sometimes fatal, effects of a culture in which a lack of compassion was the norm [11]. Since then, positive impacts of compassion on staff wellbeing and organisational health have also been identified [12, 13]. Understanding what compassionate care is, from those who seek to perform it, and how staff perceive potential aids and barriers to compassionate care in specific healthcare settings, is therefore important to patients and staff.

Whilst many systems of care are complex, involving multiple people and agencies, child and adolescent mental health service (CAMHS) wards are particularly complex and interconnected [14]. These wards provide 24-h mental health care over an average of two months, with interactions and influences among young people and their family, ward staff, managers, the wider organisation and outside agencies. Compassion is an important aspect of these relationships [15] and factors impacting on compassionate care are likely to include individual, team and organisational factors, with each level of the system interacting with the others. These interconnected systems are resonant with the idea of ecological systems influential in developmental psychology, where children are considered as nested within concentric circles of family, friends, school, other services, and wider social and political contexts [16]. In a similar way, patients are encircled by staff working with them, who are in turn encircled by their working environment, which is affected by national and international social and political influences. All levels interconnect and have the potential to affect each other in a bidirectional way. These complexities make it important to study the phenomenon of compassionate care from a ground-up perspective, and find out as much as possible from staff involved about the realities of compassionate care, its nature, aids and barriers.

A systematic review of the theoretical compassion literature by Strauss et al. [17] proposed a definition of compassion which incorporates five key elements: Recognising suffering, understanding the universality of human suffering, feeling for the person suffering, tolerating uncomfortable feelings, and motivation to act to alleviate suffering. There has been less research investigating how particular populations view compassion, or how well these theoretical models fit with grounded experiences of compassionate healthcare. Indeed, none of the previous theoretical studies have asked staff directly for their opinions on compassionate care, aids and barriers.

### Aids to compassionate care

Literature on cultivating compassion in healthcare systems, has identified a need for compassionate leadership to be present in order to foster conditions in which compassion can flourish [18], including conditions that improve patient care by enhancing supervisory support [19]. Staff wellbeing has been linked to better patient care [20], and factors that improve staff wellbeing may therefore indirectly influence levels of compassionate care.

The organisational psychology literature has highlighted the superior effect of wellbeing interventions that target multiple levels (work conditions as well as individual resilience) [20] over those that only target individuals. Potential factors that might improve both staff wellbeing and patient care include those relating to work motivation, such as “hygiene factors,” i.e. factors needed to prevent dissatisfaction (e.g. adequate supervision, work conditions and rewards), and motivating factors such as recognition and growth [21].

Additional factors linked to wellbeing include appropriate balance of work demands and individual control over how to fulfil those demands [22], adequate support including positive relationships at work [23] and staff opportunities for autonomy, relatedness and competence [24]. Factors known to help or hinder motivation have also been related to the creation of compassionate leadership and fostering of trust [25].

### Barriers to compassionate care

An important barrier to compassionate care is the stress experienced by health care professionals. Indeed, healthcare is widely acknowledged to be one of the most stressful work settings, with a report in the UK stating that approximately 40% of NHS staff had called in sick due to stress in the previous year [26]. These numbers are likely to be worse now, as the COVID-19 pandemic continues.

Stress can impair compassionate care for example by inducing compassion fatigue [27]. Compassion fatigue refers to emotional (and often physical) exhaustion and an associated reduction in ability to feel compassion [27]. It is distinct from burnout (exhaustion, hopelessness and a reduction in a sense of personal accomplishment, not necessarily associated with compassion) and from vicarious (or secondary) trauma (the triad of post-traumatic stress symptoms: avoidance, hyperarousal and intrusive thoughts, experienced in the wake of someone else’s description of a trauma) [28].

Although healthcare professionals are often highly compassionate, compassion fatigue is a particularly prevalent response to work stress in this population, possibly precisely because of how often compassion is required (and experienced) [27, 29]. Within healthcare settings, staff often have to cope with stressful and sometimes upsetting environments, tasks and interactions (e.g., in mental healthcare, helping an individual to dress their self-harm wounds, or in physical healthcare helping a patient to manage chronic pain, or delivery of a terminal diagnosis). How to manage these without experiencing compassion fatigue presents a challenge both to individuals engaged in the work and to supporting organisations.

Compassion fatigue impacts the ability of staff to provide compassionate care [20], as well as impacting service efficiency (through staff sickness or turnover) [30]. In addition, lower staff wellbeing is both a precursor to compassion fatigue, and a potential consequence, since compassion fatigue is associated with lower compassion satisfaction – the feeling of satisfaction from performing a caring role [31].

Models of how compassion fatigue develops differ in the extent to which they emphasize individual or organizational risk factors [29, 32], with the latter including factors such as poor resources and considerable patient demands. Empirical studies show an effect on compassion fatigue of both individual factors (e.g. staff history of trauma) and more systemic/organisational factors (e.g. level of support in working environment) [28]. It is therefore likely that individual, team, and organisational factors are important aids and barriers to compassionate care, but these are seldom explored from the staff’s perspective, consistent with a general paucity of studies grounded in individual healthcare providers’ experiences in specific settings.

Current knowledge about compassionate care is theoretically sophisticated but study of what compassionate care consists of, what aids it, and what hinders it is seldom rooted in lived experience. Theoretical models are yet to coalesce into an established consensus on what aids compassion and what acts as a barrier. We complement existing theoretical knowledge by examining what compassionate care means to staff working in inpatient CAMHS in the UK. We specifically focus on how these staff understand compassionate care, and what they perceive as aids or barriers to compassionate care.

## Methods

The design involved a mixed of qualitative and quantitative methods [33]. This allowed us to achieve both a rich understanding of one group of staff, and a sense of how generalisable these views were.

Initial scoping conversations with stakeholders were used to refine our research questions and approach in consultation with staff, managers and ex-patients. Stakeholder conversations informed the development of interviews for three focus groups with inpatient CAMHS ward staff from three wards, which were analysed using thematic analysis to create themes relating to elements of compassionate care, aims and barriers. Themes were explored in relation to the different levels of the healthcare system: Individual relationships between patient and healthcare professional, team factors, and organisational factors.

Once the results of these focus group discussions had been thematically analysed, we used a survey to examine to what extent these themes resonated with staff from other inpatient CAMHS services.

### Stakeholder consultation

Although the first author of this paper has lived experience working with children and adolescents in mental health services, our research started with a wider consultation with a broader range of clinicians, as well as (former) patients, to ensure our research questions and approach were relevant to the population in question. Stakeholders were selected through the first authors’ professional networks and a patient and public involvement network at Bath University, and were contacted by email or phone. Stakeholders included clinicians from a range of relevant disciplines (nursing, occupational therapy, medical, psychological, social work), mental health commissioners, and adults who had previously been patients on adolescent inpatient mental health wards. Exchanges ranged from one response to the original contact to emails/phone calls exchanged between author and stakeholder. Stakeholders were from London and the South West of the UK and were asked: (1) What does compassionate care mean to you? (2) What do you think would be useful to research in this area? (3) What in your opinion helps compassionate care to happen? (4) What in your opinion gets in the way of compassionate care?

The consulted stakeholders thought of compassionate care as an important, but as yet poorly understood, issue, deemed worthy of further research by all stakeholders consulted. The importance of empathy, kindness, and taking the perspective of another were all discussed as aspects of compassionate care. Some staff were interested in how patients feel during their first contact with CAMHS, hypothesising that the initial contact usually helps set the tone for therapy and recovery and wondering how important compassion might be at that stage. Others were interested in links between compassionate care and attachment theory, and also between compassionate care and staff burnout. Opportunities to reflect, to pay attention to self-care, and to relate theory to practice were all identified as important aids to compassionate care. Limited resources, including lack of time and poor staff relationships, were highlighted as barriers to compassionate care. Ex-patients were interested in whether patients noticed staff stress on the ward and whether patients had different views before and after admission of what compassionate care was like. A mix of people were interested in whether staff experiences with stress and compassion changed over time. All thought that different perspectives on compassionate care would be interesting to compare and that staff, if consulted, was likely to have useful ideas about what helped and hindered compassion on the ward. All were able to answer the questions meaningfully, and questions 1, 3 and 4 were used (in a modified form) as a basis for the subsequent focus groups.

### Focus groups

We next conducted three focus groups with mental health professionals on three different adolescent mental health wards in the South of England (one focus group per ward). These focus groups did not include (former) patients, as our focus in this part of the study was primarily on mental health professionals.

All wards were UK NHS services and provided care to adolescents from 12 to 18 presenting with significant distress and a diagnosis of a mental health problem. These wards did not overlap with those who participated as stakeholders in the first phase of this research. Wards had given consent for staff recruitment. A senior team member on each ward helped facilitate information sharing and recruitment for the focus group. Care was taken that there was no suggestion of coercion and that all staff voluntarily participated. All staff were invited including care staff, domestic staff, and administrative staff, although the majority of attendees were clinical staff. Staff were informed about the study through an email and participant information sheet shared with all staff by the local senior team member and through posters and participant information sheets left in staff areas of these wards. Focus groups took place in staff meeting rooms on the ward site and staff were thanked for their time with a £10 online shopping voucher.

Between 10 and 12 participants attended each of the three focus groups, with a total of 35 participants. Full demographic information was not collected but verbal report and observation revealed that groups were attended by a mix of professionals, including nurses, doctors, and other healthcare professionals (e.g. occupational therapist). Nurses were the largest staff group, reflecting the staff mix on CAMHS wards in general.^1^

Focus groups were moderated by the first author and followed a semi-structured interview schedule honed from initial stakeholder conversations. Groups lasted an hour, with three key questions: 1) What is compassionate care for you?; 2) What are, for you, aids to compassionate care?; And 3) What are, for you, barriers to compassionate care?

Group discussions were recorded and transcribed by an independent transcription service.

All transcripts were analysed together by the first author using thematic analysis [34], with data for each question analysed separately and then reviewed by the second author and by an additional postdoctoral qualitative researcher. Analyses were reflexive and informed by our own experiences as well as understanding of past research and theory. The first author has worked inpatient CAMHS settings whilst the second author and postdoctoral researcher have not.

The first author familiarised herself with the data by listening again to the recordings of the focus groups, which she had conducted, and reading and re-reading the transcripts. Raw data were reflexively coded by the first author by hand (using paper copies of the transcripts, highlighters and pen). Codes were noted in the margins and the relevant text was highlighted. Codes related to the participants’ expressions in the transcripts in both a very literal way relating to the semantic meaning of what the participant said and in some cases in a more inductive way, describing a latent meaning which seemed to be present. Codes were listed by the first author then checked by a post-doctorate researcher with expertise in qualitative analysis, and adapted in response to feedback.

Codes were assembled into initial candidate themes and sub-themes by the first author. Sub-themes were organised as clusters under each theme and the generation of sub-themes involved the interpretation of some participant experiences. Themes and sub-themes were first established in relation to each question, and then looked at overall in relation to the whole dataset. Initial themes and subthemes were then reviewed in collaboration with the second author and the qualitative expert advisor. This included consideration of whether themes or subthemes would be better conceptualised as a code, whether the themes were meaningful, useful and coherent, and whether there was enough data to support the themes. The overarching themes were mainly those that could be directly related to participants’ accounts, however, some elements of the themes drew on a priori knowledge of the first author from past theory and research (as highlighted in the introduction).Whether the data related to individual, group, or system-level experiences was also coded by the first author and reviewed by the second author. Themes, sub-themes, and naming of data as relating to individual, group or system-level experiences were modified as a result of discussions: the process was iterative. Themes were collaboratively defined and named, and a first draft of this paper produced. As a result of pre-publication peer review, themes and sub-themes were further revisited and themes were condensed further.

### Survey

After analysis of focus group discussions, we created a survey to examine to what extent the sub-themes resonated with the experiences of CAMHs workers in other inpatient services. An online survey was created on Qualtrics and shared with all members of the Quality Network of Inpatient CAMHS (QNIC). This is a mailing list run by the Royal College of Psychiatry that staff working on inpatient mental health units for children and teenagers (from multiple professional disciplines) in the UK and Ireland can choose to join. Network members were invited to participate in the survey; anonymity and the voluntary nature of participation were stressed.

Data was collected for six weeks and three reminders were sent within this period. The survey received 36 responses. Nearly 70% of respondents worked in adolescent inpatient units. Of the 36, 25 (69.4%) worked on adolescent mental health wards, three (8.3%) worked on child wards, two (5.6%) worked on adolescent Psychiatric Intensive Care units, and six (16.7%) worked on other types of child and adolescent mental health wards. Roles were allied health professionals (AHP) (*n* = 12, 33.3%), nurses (*n* = 8, 22.2%), doctors (*n* = 5, 13.9%), Health Care Assistant (HCA) (*n* = 1, 2.8%) and other (*n* = 9, 25.0%); one participant did not provide information about their role.

Participants were asked how much they agreed that each of the sub-themes reflected their experiences in relation to each question. For example, participants were asked to what extent “active listening” was inherent to their understanding of compassionate care and to what extent they experienced “self-criticism” as a barrier to their ability to deliver compassionate care. Participants responded to these questions on a six-point Likert-type scale (from 0 = not at all important to 5 = extremely important). Participants were then asked whether they felt any factors were missing from the list presented, and if so, to describe these.

## Results

### Focus group findings

Themes and sub-themes are presented in Tables 1,2,3 and summarised with example quotes. There were no noteworthy differences across focus groups at different locations.

**Table 1:** Elements of compassionate care (Level: I, individual; T, team; O, organisation)

Theme	Sub-theme	Level
Emotional Connection	Empathy for the patient	I
	Helping the patient feel safe	I,T,O
	Sitting with difficult feelings	I
	Therapeutic touch	I
Sense of being valued	Prioritising patient care	I,T,O
	Having a non-judgemental attitude towards the patient	I,T
Attention to the whole person	Active listening	I
	Individualising care	I,T
	Spending time with the patient	I
	Working as a collective around the person	T
Understanding	Making sense of what is happening	I,T
Good Communication	Collaborating with the patient	I
	Explaining what you are doing	I
	Showing the patient that you care	I
	Normalising distress	I
	Authenticity and openness	I
Practical help/resources	Enabling patient independence	I,T
	Offering practical help	I

Responses to the first question (What is compassionate care?) were categorised into 6 themes with 18 sub-themes (see Table 1), including practical actions that demonstrate compassionate care and attitudes that staff hold as part of compassionate care. Overarching themes were: Emotional connection, sense of being valued, attention to the whole person, understanding, good communication, and practical help/resources. In all tables below, the level of system to which themes related is indicated by I, T, or O (individual, team and organisation).

*Emotional Connection* with young people on the ward was seen as a crucial part of compassionate care that underpinned further work, and included: “Get[ting] to know them first” (Healthcare assistant, ward 2). This theme was sub-divided into empathising with the patient, helping patients to feel safe, sitting with difficult feelings and therapeutic touch. Empathy was mentioned by several staff. Staff spoke about “tuning in” to how young people on the ward were feeling (Doctor, ward 2), relating to those feelings “as a human being” (Nurse, ward 2), and showing young people that “you’ve seen how they feel” (Nurse, ward 1). A balance between really feeling for the person, but without becoming overwhelmed yourself was described: “You’re not there in the hole with them but you… see where they’re coming from” (Healthcare Assistant, ward 2).

Helping the patient to feel safe was described both as reassuring them through words and actions, and through staff modelling safety through their behaviour with each other: “They trust us ‘cause they see us look after each other” (Nurse, ward 1).

Sitting with difficult feelings was described by one nurse as “Sitting with them when they’re distressed and it doesn’t matter how distressed they get you’re still there.” One staff member related that those difficult feelings could even be brought up by receiving compassion, if this was an unfamiliar experience: “When they experience compassion it can sometimes bring up times when they might not have had that experience in the past. It can bring up a lot of painful feelings for them so being able to sit with them through that” (Nurse, ward 1).

Several ward staff also felt that the willingness to touch the patient, appropriately, was an important part of compassionate care. This could take different forms, either “touching a hand on the shoulder” (Nurse, ward 2) or getting down on the same level as a distressed patient and holding their hand, or offering to help plait patients’ hair: “That hands on approach that we care enough to do it” (Healthcare assistant, ward 2).

A *sense of being valued* was subdivided into sub-themes of prioritising patient care and having a non-judgemental attitude towards the patient. Prioritising patient care was common to staff’s understanding of compassionate care. One senior nurse spoke about prioritising face-to-face contact: “I spend a lot of time doing paperwork but if someone is in need of my care and my compassion then I make time for that” (Nurse, ward 1). Conversely, one staff member spoke about how prioritising patient care could take many forms, which were not all witnessed by the patient themselves but which did all involve providing compassionate care:

“It’s easy to get drawn into feeling that the only time we’re being compassionate is when we’re sat with somebody and soothing them… they don’t necessarily see my compassion when I’m shouting at a social worker down the phone because a placement hasn’t been found” (Nurse, ward 1).

Suspending judgement about young people’s behaviour was an important aspect recognised by several staff members: “Being non-judgemental, making sure that… if you’ve got your own beliefs not letting that affect your ability to care for the person that’s in front of you” (Nurse, ward 2).

*Attending to the whole person* included active listening, individualising care, spending time with the patient and working as a collective around the person. Many participants described qualities of active listening, and reasons for why this was so important for compassionate care, including the more practical seeking of patient views and the more emotional “responding to somebody’s feelings”. For example:

“Normally you try to reflect back what they’re saying or give a little summary if they’ve said an awful lot. So they know that you’ve heard what they’re trying to say. Or if you get it wrong they’ve got the opportunity to say no, no, not like this, it’s like this” (Nurse, ward 3).

Whilst ward rules and boundaries were viewed as important there was also a recognition that to provide compassionate care sometimes rules need to flex to accommodate individual needs. Sometimes this included flexing the rules to allow increased comfort, for example around letting young people have teddy bears with them through the day, or letting them “curl up under a blanket” if they were having a difficult time: “Tailoring care to individual circumstances… otherwise patients can merge into one big patient we’ve seen many many many times… to press pause a moment and think about that individual” (Nurse, ward 1).

To provide compassionate care, the importance of spending time with patients was emphasised, even when this was not always to do anything in particular, but more about sitting with someone: “Just staying in the room. Just sitting there quietly sometimes” (Nurse, ward 2).

Working as a collective around the young person with other staff members and the young person’s family was seen as an important aspect of compassionate care in the ward setting in order to bring in different aspects of their lives: “It really encapsulates everyone that’s involved in that person. If you’re all compassionate and work as a sort of collective…everybody including their folks, and carers, or social services… that big circle around them” (Nurse, ward 2).

Relatedly, seeing the staff team as one entity in which different team members could swap in and out of working with a young person was another strong theme: “Sometimes it’s recognising that actually, I’m not getting anywhere here, maybe someone else might be able to help” (Nurse, ward 3).

*Understanding* was an important overarching theme. Staff members talked about making sense of young people’s feelings and behaviours both as a way to promote their own (staff) ability to care compassionately “understanding their behaviours instead of just pushing them aside” (Nurse, ward 2), and as a way of helping young people to understand what might be going on for themselves: “Support the young people to see why we’re doing what we’re doing” (Nurse, ward 3). An exception was one medical staff member who disagreed, and didn’t think understanding was needed to feel compassion; He stated: “you can have compassion for someone’s emotions without understanding why they feel that way…. I don’t necessarily understand it but I can see it and I want to help you” (Doctor, ward 2).

*Good Communication* consisted of collaborating with the patient, explaining what you are doing, showing the patient you care, normalising distress, and embodying authenticity and openness. Collaboration with patients and families included actively seeking views, collaboratively setting goals for admission, behaving in a way that patients have requested (e.g. following agreed care plans), and checking plans out before putting them in place. To illustrate, one nurse stated: “Young people have lots of things done to them, so [this helps us to] trying to get more alongside” (Nurse, ward 1).

Being able to explain clearly what was going on and why, and to offer alternative options, was also felt to be an important part of what staff felt qualified as compassionate care. This included thinking about how to explain if something that the young person wanted to happen couldn’t happen: “If you can’t do what they’re asking explaining why and perhaps offering to do something slightly different” (Nurse, ward 3).

Staff spoke about the importance of showing you care, “being explicit that you care” (Nurse, ward 1). One nurse said: “I think it’s important to let them see that you care… okay you’re telling me to go away right now but actually I’m really worried about you and I care about you so I don’t want to leave you” (Nurse, ward 1).

Staff spoke about the importance of normalising distress, understanding and empathising with distress and wanting to help, as you would help anyone who was struggling. One nurse summed this up as helping patients to understand for themselves that: “You do things differently when you’re having a bad day” (Nurse, ward 1).

Staff emphasized the importance of openness about the impact of caring on themselves, even when this might involve more difficult conversations, for example letting a patient know that something they have done has made the staff member worried or concerned. This also related to authenticity. This was spoken about as an important aspect of care, so that patients weren’t receiving mixed message. In this sense, being compassionate involved “Being open, not saying one thing but actually they’re picking something else up” (Nurse, ward 1).

*Practical help/resources* was a theme spoken about by multiple staff in relation to the importance of empowering patients, providing them with new skills, helping them to see different ways of doing things and encouraging independence rather than “rescuing”: “When you help someone by not helping them… not doing everything for them” (Nurse, ward 3).

Several examples of practical help were given to illustrate compassionate care, both for patients (e.g. “helping them to put on their shoes” Nurse, ward 2, or “making them a cup of tea” Nurse, ward 3), and for family members (e.g. “offering them a box of tissues” Nurse, ward 3). The importance of “something small you can do to make them feel more comfortable” (Nurse, ward 3), often going beyond baseline clinical care, was a common theme, as shown by the following example:

“We had a parent who was in a wheelchair and really restricted mobility and I recognised that her young person’s gone for something to eat and I was on my way out to get some lunch, and I just stopped and said “is there anything I can get you?”, because I knew the likelihood was she couldn’t… She asked me to get her something and that was just such a little thing to do for somebody, but I thought ‘I’m really glad I did that because she’d have been really hungry’” (Nurse, ward 3).

Responses to the second question (i.e., what might aid or facilitate compassionate care) yielded six themes that were similar to those obtained in response to question one, with 18 sub-themes. However, this time, the 18 sub-themes involved individual factors related to the staff members and their ways of coping with work, team factors, and factors related to the organisation as a whole, including the actions of other members of the system, e.g. colleagues, senior management, and young people. Themes and sub-themes are summarised in Table 2.

**Table 2:** Aids to compassionate care (Level: I, individual; T, team; O, organisation)

Theme	Sub-theme	Level
Emotional connection within system	Compassion of other team members	T
	Feeling cared for	T,O
	Connection with staff team	T
	Connection with patient	I
	Team work	T
Sense of being valued	Feeling valued	I,T,O
	Celebrating successes	T,O
Being attended to as a whole person	Training/Personal Development	O
Understanding	Formulation	T
	Thinking space	T
	Individual supervision	I, T
	Non-judgement	I,T
Good communication	Respect for different points of view	T
	Team thinking/talking	T
	Authenticity and openess	I,T,O
Practical help/resources	Time away from ward	I,T,O
	Coping strategies	I
	Adequate staffing resource	O

*Emotional connection with the system* was seen as an aid to provision of compassionate care. This related to how caring the organisation felt to the staff working in it, and how emotionally sensitive other team members were perceived to be. Compassion shown team members was described as “infectious” (Nurse, ward 2). Part of this was related to modelling compassion so new staff starters could see how it was done. This included staff being kind to each other when mistakes were made: “If people make a mistake, it’s okay, people recognise you made the decision you thought was best at the time” (Nurse, ward 3).

Examples of feeling cared for at work were given by multiple ward staff. Being held in mind by the staff team as a new starter, having people notice if you are struggling, being able to talk to colleagues about professional and personal dilemmas, and having basic physical needs looked after by other staff, all were given as important examples of feeling looked after by colleagues through the stressful events of day-to-day ward life:

“You have a really rubbish hour and someone will have gone on their break and bring back the most doughnuts you’ve ever seen” (Nurse, ward 1).

Part of feeling cared for by a compassionate team was a feeling that other team members would be attuned to staff distress and act to do something about it, without a staff member necessarily needing to ask for help. For helping professionals used to caring for others this is perhaps particularly important: “We’re good at spotting when someone is struggling. I’d say we’re stronger at spotting when someone is struggling than we are at actually asking for help” (Nurse, ward 1).

Many staff members spoke about the importance of a strong connection with the wider staff team, which encompassed multiple practices of building and maintaining this connection, including small gestures such as smiling at each other in the corridor, and more difficult practices such as making sure repair happened after difficult conversations between staff: “Everyone’s going to fall out [staff]… but it’s what you do next to repair that is valuable” (Nurse, ward 1).

One staff member highlighted the important of handover as a way of facilitating this connection, encouraging an understanding of the events of the last shift better and promoting compassion for the staff and young people who may have been through some difficult experiences in that time. Another emphasised the idea of the ward as an entity involving multiple people – staff, patients, and families – and one where care is continual, even when the particular staff member is not on duty: “It’s something about being connected to the whole, ‘cause it’s 24/7” (Nurse, ward 1).

Establishing and maintaining connection with the patient was also seen as an aid to compassionate care. Some staff spoke about feeling that a patient had a particular resonance with them, as if “the patient tends to pick you” (Nurse, ward 2), whilst others found they felt more empathy for particular types of mental health difficulty: “I probably get on more with people who have psychosis rather than mood disorders. I find with mood disorders I probably end up going down in the ditch with that person whereas with psychosis I can stay there” (Nurse, ward 2). Knowing about an abusive history was spoken about as something that was likely to increase staff compassion for that young person for most staff.

Staff also saw team work as an aid to them remaining emotionally fit enough to care in a compassionate way. Teams were often described as acting “like a family” (Nurse, ward 1), with team members being supportive and taking time to give practical help when needed: “if something is going on on the wards, you’ll find everyone is down on the ward doing what we can” (Nurse, ward 1). Another participant stated: “We have those squabbles, but that’s okay because we all care about each other enough to make up afterwards and still continue to work together in a really collaborative and cohesive way” (Nurse, ward 1).

*Feeling valued* was a theme that related to experiences which gave staff a sense that people valued their work, whether that was colleagues, managers or patients and families. Many staff spoke about how important this was to their sense of connection to the ward and their ability to be a compassionate carer, as well as to a sense of feeling nourished by the work rather than depleted. Multi-disciplinary teams in CAMHS ward settings mean staff do different roles, and one feeling was that this meant that different roles “value the stuff that they can’t do, that we can, and vice versa” (Nurse, ward 3). The way staff showed value to one another, from the most senior down, in small gestures such as remembering your role and name, was also deemed important.

The importance of patients valuing work the staff had done was clear. Staff remembered being contacted by some patients years after having cared for them:

“Sometimes they write lovely letters about how much they feel that the ward has helped them or sometimes, even now, somebody who left about a year ago will phone up and say this is happening, or I just got my GCSE results or whatever. It’s lovely. You don’t want to keep them connected with us necessarily but at the same time you know your work has been valuable to them” (Nurse, ward 3).

This fitted with the idea of how important it was to celebrate successes, in order to keep staff feeling motivated to care compassionately. This related both to staff and patient successes, for example from small acknowledgements of a positive piece of work to opportunities in shift handover to speak about young people with optimism: “Looking at something positive that they’ve done and the way you speak about it… helps the staff when they go onto the ward… you’ve got more compassion when you’re dealing with them” (Nurse, ward 2).

*Being attended to as a whole person* was valued by staff as a way of enabling them to be able to take on their caring role, just as it was identified in the first question as an aspect of compassionate care that was important for young people. Staff noticed the importance of being able to be honest and authentic about their feelings even if they were difficult: “thinking that’s okay sometimes to feel really angry or feel really bitter and not feel that compassion… not putting yourself down helps it to come back again… allowing yourself to feel horrible for a bit because that’s normal” (Doctor, ward 1). For staff, training and personal development, both in terms of formal training and learning from peers through watching them (“I will try to do what they’ve done” (Nurse, ward 2) and through discussion, were identified as aspects of the work which were nurturing and allowed increased compassionate care. Training in compassion was valued and remembered as part of the staff induction in one Trust, but learning about compassion through seeing colleagues respond seemed to be valued more: “There’s nothing worse if it’s death by PowerPoint… I’d much rather talk about it that read it off a slide but also there’s something about learning from somebody else…” (Nurse, ward 2).

*Understanding* was thought to help compassionate care in a range of ways, many of which were to do with encouraging understanding of the patient’s problems. Making time for thinking space in order to increase understanding included debriefs, informal 1:1 s, individual supervision and formulation, and some of these aspects were also discussed separately and named as particularly important. All involved opportunities for discussion with colleagues in order to understand and maintain compassion towards patients, especially (but not only) after incidents on the ward such as an assault of a staff member by a young person: “Having that space to formulate and take a step back and think actually what is compassionate for this person at the moment…” (Nurse, ward 1).

Individual supervision from more senior colleagues was talked about in particular as something that was vital, in order to “see beyond” (Nurse, ward 1) what was going on and retain compassion: “Being able, through supervision, reflection and all these other things, to be able to see beyond perhaps what you’re faced with” (Nurse, ward 1).

Formulation, consisting of a shared understanding within the team (and with the patient and their family) of what is going on for the young person, was talked about as being important for staff to have a shared knowledge framework, but also as a way to grow and sustain compassion: “If you understand the reasons why someone’s behaving in a certain way that helps you feel more compassion” (Doctor, ward 2).

*Communication* was identified as an important aid to compassion, both time to discuss team dilemmas either directly related to clinical care or not, and open and respectful communication between team members. Encouraging respect for different points of view enabled multiple perspectives on a young person’s care to be thought through in order to encourage compassion and also helped staff to feel that they were understood and viewed compassionately by the wider team, regardless of hierarchy: “Everybody’s opinions matter” (therapist, ward 1).

Authenticity and openness were named by some staff as important in promoting compassion because they enabled staff to relate more authentically to each other and to patients, and to appropriately feel their own feelings rather than “keeping a lid on everything”.

A senior nurse described: “I’ve named it with the young people only this morning. I’ve had three of them in a room and I’ve said ‘I’m really disappointed, I’m angry, I’m upset with the way you’ve treated this ward’, because it’s role modelling, we’re allowed feelings” (Nurse, ward 1).

Relatedly, holding a non-judgemental attitude towards oneself as a staff member was deemed important for staff to authentically cope with the difficulties of the role, accompanying the ability to be compassionate or to return to compassion if for a while it felt absent: “to be compassionate to yourself not only the patients” (Nurse, ward 3). One nurse said: “Thinking that’s okay sometimes to feel really angry, or bitter, or not feel that compassion. Not putting yourself down… just helps it come back again” (Doctor, ward 1).

*Practical help/resources* included a range of strategies to cope with the emotional toll of working on the ward and these were seen as important in enabling compassion. Named strategies included dark humour amongst the team, making sure they have time to do something after work that is just for them (examples ranged from gardening to drinking whiskey), being able to “have a bit of a rant” about emotions such as annoyance, amongst the staff team, being able to tailor workload so it is more manageable or more varied and taking time off the ward. One participant stated: “You can’t pour from an empty cup so you’ve got to top yourself up before you can really properly give to other people” (Nurse, ward 1).

Taking time off the ward in different ways was a prominent theme. One ward had renamed staff breaks as “patient safety breaks” (Nurse, ward 1) to emphasise the impact they had on patient care as well as staff wellbeing. Alongside this was a recognition that it was better to communicate to management if you were struggling to manage emotionally and take some time off the ward, if this was a possibility: “When you’re not quite right… have the afternoon at admin” (Nurse, ward 1).

Adequate staffing was one aspect of practical resource, and “Not being short staffed” (Nurse, ward 2) and “having time to check in with the children [patients]” (Nurse, ward 2) were seen as directly related. Thinking carefully about the number and role of the staff present on different shifts was also seen as important, as different times of day on the ward have different demands. For example, the early evening on CAMHS wards is a time routinely associated with increased risk incidents such as aggression or self-harm and is a time when there are fewer structured activities and fewer non-nursing staff. One ward introduced a twilight shift (between 4 pm and midnight) to increase staff at this time and found this reduced incidents. A manager spoke about the importance of having a higher level management presence on the ward at times:

“S--- higher level manager is here once a week so she’s always around if you ever feel like you need to go to a higher level…. That felt supportive and enables you to work better because you really feel listened to because the top people are out talking to you” (Nurse, ward 3).

### Barriers to compassionate care

Responses to the third question (i.e., what might be the barriers to compassionate care) again reflected six themes that were similar to those observed in response to the first and second question, this time subdivided into 19 sub-themes. These were again related to different levels of the system: individual factors, factors related to team relationships with other colleagues, and organisational factors. Themes and sub-themes are summarised in Table 3.

**Table 3:** Barriers to compassionate care (Level: I, individual; T, team; O, organisation)

Theme	Sub-theme	Level
Poor emotional connection	Constant change	O
	Prescriptive rules	T, O
	Lack of clear boundaries	T,O
	Lack of connections with team	T
	Impact of patient presentation	I
Low sense of value	Lack of reward	I,T,O
	Poor work conditions	O
Lack of attending to the whole staff member	Challenges in personal life	I
	Impact on home life	O
Lack of understanding	Lack of improvement in patient	I
Poor communication	Relationship with management Lack of authenticity	O
Lack of practical resources	Unhelpful personal coping styles	I
	Feeling worn out	I
	Lack of practical support	O
	Lack of external resources	O
	Short staffing	O
	Physical ward environment	O
	Lack of time	O
	Bureaucracy	O

*Poor emotional connection* with the healthcare system as a whole was described in relation to constant change and inappropriate or inflexible rules, which they also saw as barriers to providing compassionate care.

Constant change in the way services were run was highlighted by both managers and frontline staff as a barrier to being able to provide compassionate care, since staff were preoccupied by acclimatising to changes. Teams were described as particularly sensitive to any kind of change because of the amount of changes that had already occurred, in quick succession, and with a lack of consultation. “That one change is probably not going to be seen as a positive change because people are still disgruntled about everything else… you can’t plonk a house together… it’s brick by brick. One change at a time” (Nurse, ward 2).

Ward rules were seen as important. There was a sense from some staff that “blanket rules” (Nurse, ward 3) on the ward were unhelpful and acted as a barrier to more individualised care, which staff felt was intrinsic to compassionate care. Conversely, a lack of rules and boundaries was also seen as unhelpful:

“Lack of consequences for actions sometimes… I’m not saying we need to punish young people in CAMHS inpatient units but that feeling that they can do whatever they want and we don’t have any power to put a consequence in… and boundaries, that can then make it harder to remain compassionate” (Nurse, ward 1).

Lack of emotional connection also related both to a lack of connection with fellow team members and a lack of connection to the young people being cared for.

Staff generally did not feel a lack of connection with the wider team, but they did feel this was an important barrier when it did occur: “If somebody’s said something to you and you’ve taken it quite personally and critically and you’ve just had a big flop then… if you perceive somebody else being a bit mean to you that can really have an impact on your ability to be compassionate to others” (Nurse, ward 1).

When a lack of connection with a young person was perceived this was also seen as having a negative impact on compassion. Sometimes this related to specific aspects of patient presentation, and this was often related to young people continuing to hurt themselves or others: “It’s really difficult when someone keeps trying to hit you” (Nurse, ward 3). Staff also spoke about how feeling that they couldn’t ‘get through’ to a young person could block their ability to feel compassion as strongly: “some of them have got walls up” (Nurse, ward 2) A feeling that a patient didn’t need to be on the ward, perhaps because their behaviour was seen as less related to mental health problems, was also seen as a barrier to compassion: “If we feel that maybe they’re not poorly anymore then that’s harder to keep the compassion for them” (Nurse, ward 1).

The potential for staff traumatisation in events such as when restraints are used, was also highlighted as an important factor that could jeopardise connection with a patient and block compassion: “Things like restraints… you’re holding someone down who is screaming in your ear and it echoes right in there and it can stay there” (Nurse, ward 2).

*Low sense of value* was discussed explicitly in terms of a low sense of reward from the work, and more implicitly in terms of the work conditions which staff experienced.

Staff mentioned a sense that not feeling rewarded by the way that they were continually asked to do more by the organisation without full acknowledgment of what they were already doing could be wearing: “It just grinds slowly, slowly, slowly… we should have done this, or we should have done that” (Nurse, ward 2).

Poor working conditions for staff on the ward were seen as an important, and under-acknowledged, barrier to compassionate care: “the team has to feel looked after” (Nurse, ward 2). Staff referred, for example, to not being provided with tea, coffee, and milk, and having to bring their own if they were to have some during the day. This led to staff feeling undervalued (and to a wasteful amount of milk in the fridge) and added to a sense of exploitation: “Instead of telling me to be positive they should be giving me more money or free coffee” (Nurse, ward 2) There was a definite emphasis on how important is was for the team to feel looked after: “Getting those basic needs met and then you’re able to perform and give that 100% and be compassionate” (Nurse, ward 2).

*Lack of attention to the whole staff member* was a theme that related to the staff as a person with both personal and professional needs and aspects to their life. Staff acknowledged the impact of their personal life on their work persona, and the difficulty in maintaining compassion, “if you’re just having a really bad time personally” (Nurse, ward 1). Conversely, the impact that working in a CAMHS ward setting could have on home life was seen as a potential difficulty: “It’s difficult to have your life here and have your life there [home].” (Nurse, ward 3). This was seen as a something which could impact on staff’s feelings about work and patients, making them less able to be compassionate with young people*.*

*Lack of understanding* was related specifically to lack of change in the patient. A common theme was that of the difficulty staff had in maintaining compassion when they felt a young person wasn’t responding to treatment, or behaving in ways that (though they might be part of their condition) make recovery harder. One nurse stated: “When people aren’t getting better” (Nurse, ward 1) and another said “when they’ve self-harmed or hit out or ran away… and that is really demoralising” (Nurse, ward 1).

*Poor communication* between ward staff and more senior management staff was seen by some participants as a barrier to provision of compassion, when there was a “disconnect between seniors and what goes on the shop floor” (Nurse, ward 1). Managers were described as often quick to want something done, but slower to respond to the needs of the ward, and on one ward a sense of being monitored was felt to be unhelpful and negatively impact on staff and their ability to care: “There’s a lot of scrutiny from senior managers looking in at this place… which can be tiring, quite exhausting and annoying” (Nurse, ward 1). This was seen to relate to senior management not fully trusting staff with information and with a more general lack of openness from more senior levels of management: “if they were open you would… understand timeframes and help them to take longer or faster” (Nurse, ward 2).

A lack of openness in staff was related to a self-critical attitude which they identified, and which they then related to coping by hiding their true feelings (and being inauthentic). Staff spoke about failing to be compassionate with themselves: “We struggle to have compassion for ourselves… we need to call our own breaks ‘breaks for the patients’ because we just cannot accept that we could possibly need any care ourselves,” (Nurse, ward 1). Staff acknowledged that these strategies were risky: “Burnout happens really quickly when the inside doesn’t match the outside for some reason” (Nurse, ward 1).

*Lack of practical resources* related both to a lack of individual resources and to a lack of practical support. Staff identified the impact that their own thinking and behaviours towards themselves could have, in particular self-critical attitudes meaning that it could be hard to show compassion if they were “not feeling good enough” (Nurse, ward 1) at the job themselves: “You might be thinking ‘I should have done that’ when nobody else is thinking that at all” (Nurse, ward 2).

Several staff acknowledged that they needed to be more deliberate about taking care of themselves and that often staff coped in unhelpful or counter-productive ways, like engaging in excessive drinking. As one team member said: “We’re human as well and we have our own mental health to look after… a lot of the time we don’t do it very well” (Nurse, ward 2).

Staff described the consequence of a lack of personal resource: Feeling “worn out” or “at your wits end” (Nurse, ward 3), as a barrier to compassionate care. This is often defined as one of the components of compassion fatigue, and was identified as something which led to a need for self-protection, but also as something that could result in feelings of guilt “after a while we will protect ourselves but we’ll feel bad” (Nurse, ward 3). One staff member stated: “It’s not that you don’t care, but it’s just where you’ve got to the point where you’ve got nothing left to give” (Nurse, ward 3).

Lack of practical resources in terms of the support which staff received from the wider system related to many sub*-*themes, including physical ward environment, provision for staff basic needs, lack of time (also related to burden of bureaucracy) and a lack of external resources and staff. Many of the factors included in a lack of practical resources could also be conceptualised as poor working conditions.

Several physical features of the ward environment were described by staff as a barrier to being in a state of mind, in and outside of work, which is calm and compassionate. Alarms going off on the ward was one feature that seemed to particularly stress and stay with staff, even when they weren’t on shift: “I was at Lidl and I literally took a step [when an alarm went off] and my husband’s like What?… I just literally thought it was these alarms” (Nurse, ward 2).

Staff described their basic needs not being met at work, for example having time to eat, use the bathroom or rest, and this impacting on their ability to provide compassionate care: “things are so difficult when you’re so tired, when you need the toilet, when you’re hungry, it’s all the basic things isn’t it? They massively get in the way” (Nurse, ward 1).

The amount of bureaucracy, including forms that need to be filled in multiple times, was identified as a barrier affecting nurses in particular: “the amount of paperwork that qualified nurses are expected to do and the duplication of that frustrates me” (Nurse, ward 1). There was also a related theme about the impact on the team of the sorts of questions asked in fact-finding or incident-related paperwork: “I don’t know if the system always encourages us to be compassionate towards each other. I think the system and the paperwork is often about placing blame or saying that things need to be done…when actually it was a really hard shift and it could get done next shift” (Nurse, ward 2).

The trade-off between time spent completing paperwork and time spent with patients was a common theme: “If they [nurses] had less red tape they’d be able to spend more time [with patients]” (Nurse, ward 1). One healthcare assistant described having to reduce attendance at therapeutic groups “it’s just managing that one with the time” (Healthcare assistant, ward 1).

A lack of financial resources available to the service was highlighted as a practical barrier to being able to provide compassionate care, particularly when staff had to be creative to meet patient needs: “They’ve taken away the money [for activities with YP] at the weekends… our consultant used her own money to buy pizzas [for patients]” (Nurse, ward 2).

Short staffing was another example of a practical barrier to providing compassionate care: “We were short staffed for a bit and that was really hard… bouncing from one incident to another” (Nurse, ward 2).

### Findings from the follow-up survey

Results from the survey (Supplementary Tables S1,S2,S3) indicated that all sub-themes listed were endorsed by at least one participant and the importance of compassion running throughout all levels of the organisation was highlighted by open comments, such as: “from board to ward, staff need compassion too.” Most sub-themes were rated as at least somewhat important or above (i.e. a rating of 2 or above on the 0–5 response scale) and seven were rated by all respondents as very or extremely important (a rating of 3 or above on the 0–5 scale). These were: Having a non-judgemental attitude and empathy as elements of compassionate care, and having the aids of respect for other points of view, feeling valued, teamwork, formulation, and adequate staffing resources. Five sub-themes were more weakly endorsed than the rest, with some staff voting them as not important at all. These were: Therapeutic touch as an element of compassionate care, authenticity as an aid to compassionate care and personal challenges, coping styles, and impact on home life as barriers to compassionate care.

When asked what factors might be missing from the list presented in the survey, some ideas were mentioned, but they all seemed to fit within existing overarching themes, though they did add detail. When commenting on what compassionate care consisted of, participants mentioned an understanding of the biology of stress and trauma and an understanding of the spirituality of young people being cared for. Both these factors can be seen to fit under the theme of ‘understanding,’ adding elements to what staff feel they must understand to provide compassionate care. For aids to compassionate care, participants mentioned the inclusion of families as an additional aid, which can be seen to relate to understanding, helping and communicating, and the recognition of team work difficulties was related to connecting with the team. For barriers to compassionate care, participants added lack of team conversations, especially about compassion, a culture of staff fear of complaints, a “double-edged” nature to teamwork and a tendency for staff to make assumptions about families. These can be seen as related to poor emotional connection within the system.

## Discussion

Our goal with this research was to shed light on what staff working in CAMHS inpatient wards in the UK perceived as elements of compassionate care, as well as what they perceived to be aids or barriers to this type of care. After consulting relevant stakeholders and refining our questions and approach, we conducted three focus groups with a total of 35 participants from a range of disciplines and roles to establish key elements of compassionate care, aids and barriers. Quantitative data from a survey with employees from other CAMHS inpatient services, also in the UK, confirmed that the sub-themes were important to them too and revealed no additional themes.

The six core elements of compassionate care identified by staff could be grouped into six themes around emotional connection, a sense of being valued, attention to the whole person, understanding, good communication, and practical help/resources. These staff perceptions of the nature of compassionate care relate to wider literature on the nature of compassion, and extend previous models to include some new and more specific elements, such as: valuing, attending to the whole person, and increasing practical resources. Staff-identified aids and barriers to compassionate care mirrored each other and related to the same seven higher-order themes they described as being important for compassionate care. In a nutshell, staff needed many of the core elements as what they perceived patients to need. For example, providing practical resources for young people on the wards was seen as an element of compassionate care, and the presence of practical resources for staff was seen as an aid to being able to provide compassionate care. Conversely a lack of practical resources for staff was identified as a barrier to being able to provide compassionate care.

The aids and barriers that were identified did not fit with any particular model of organisational wellbeing, although elements overlapped. For example, the importance to staff of being valued, having a good connection with colleagues and having the practical support to be able to do their role well, fits with several theoretical models of work wellbeing and motivation [21, 23, 35].

Factors that staff described as important for compassionate care, and which related to aids and barriers to providing this care, reflected the nested systems in which ward staff work, from the individual patients, to the clinical team, to the broader organisation. A ward is an interconnected system in which young people, ward staff, managers, and the wider organisation interact and influence each other. Individual, team, and organisational factors were identified as important in enabling, promoting and preventing compassionate care. Staff experiences of being able to care for patients and be cared for by patients, colleagues and the organisation at large showed the interconnectedness of the systems involved and highlighted the importance of looking after the staff who look after patients and families. Some of the elements of compassionate care identified are resonant with literature on therapeutic relationship [36] and it is likely that there are overlaps in the constructs.

In general, elements that staff identified as part of compassionate care for patients related more to the individual relationship between staff member and patient (e.g. active listening), although a minority of sub-themes related to team input and to organisational context as well (e.g. helping the patient feel safe). Staff-identified aids to compassionate care were a mix of individual factors (e.g. helpful coping strategies for managing stress at work), team factors (e.g. feeling connected to the staff team) and organisational factors (e.g. feeling valued at work). Barriers to providing compassionate care identified by staff related mostly to organisational factors (e.g. lack of resources, lack of practical support) although some individual factors (e.g. difficulties in personal life) and team factors (e.g. lack of connection with the team) were also identified.

The multi-level staff perceptions of what compassionate care is and how it is aided and prevented fit with the idea of the importance of nested systems from developmental psychology [16] and with the literature both on cultivating staff wellbeing by influencing multiple levels (organisational as well as individual) [20] and on using leadership to impact on compassionate care in healthcare systems [18].

It is notable that whilst individual coping strategies were identified as important by staff, both as a tool to provide to patients and for staff to have access to, staff emphasised these less than other factors. This is in contrast to the current emphasis on individual-level interventions for individual resilience to work stress, coping with stressful environments, and positive rather than unhelpful coping styles, which are often the focus of trainings for staff and patients [37]. This could represent a gap in interventions, which tend to target individual level rather than organisational level factors, and could also indicate a gap where psycho-education (e.g. wellbeing training) could be helpful for this participant group, if they are unaware of the evidence available on more individual coping styles. It is likely that both psycho-education and multi-level interventions would be helpful.

Our findings also relate to literature on compassion fatigue [27]. They support a broader model of compassion fatigue as relating to both individual and organisational demands and protective factors. Specifically, they support Figley’s [29] inclusion of life demands as a risk factor for compassion fatigue, and of sense of satisfaction as a protective factor, and they support Coetzee and Laschinger’s [32] inclusion of resources, patient demands, ability to reflect, feedback and ability to replenish resources, all of which were mentioned by staff in this sample.

Whilst this study provides an important staff perspective in inpatient CAMHS settings, some limitations are notable. Other important points of view are missing, especially those of patient and family members/carers. Patients and family members also have views that are relevant for these questions, especially with regard to what constitutes compassionate care. They might also have views on what might be barriers and facilitators of this care, particularly on how they might contribute to hindering or facilitating their experience of the care they receive as compassionate, or what they believe staff can do to express their compassion in clearer and more effective ways. More research is needed on other perspectives, including patients, families, managers and commissioners, to more fully understand care in this setting.

The quantitative study was also limited by the modest number of respondents, despite significant efforts to increase participation, so future research could aim to recruit larger numbers of CAMHS ward staff to further assess the generalisability of our findings. In addition, the results in this study capture views at one time point, so future studies could explore whether staff attitudes towards compassionate care change over time, for example as their professional experience accumulates, or as policy changes occur. In addition, we did not focus on gathering objective evidence of barriers and facilitators to compassionate care, but on how staff perceive these. Although subjective perception is likely to be a crucial mediator between real conditions and staff wellbeing, future research might focus on triangulating perspectives, or on examining longitudinally the effects of the factors staff identified as aids or barriers to compassionate care to identify whether or not they ultimately function as such. Finally, we did not gather demographic information about our focus group participants which has prevented any observations about trends relating to demographic variables.

## Conclusions

Our findings shed light on what staff themselves aim for when they aim to deliver compassionate care. As such, they can be used in training and by service managers to understand staff’s perspective on their work. The findings regarding aids and barriers may be particularly helpful as a basis for future work testing the influence of the identified factors on staff’s ability to deliver care that is perceived (by them and or patients) to be compassionate and effective. Ultimately, these findings can contribute to the development of interventions aiming to improve compassionate care and prevent/reduce compassion fatigue. They support the importance of a multi-level approach to promoting compassionate care and the idea that interventions that impact on staff care and wellbeing need to address nested systems [19].

The findings add to the literature on compassionate care in child and adolescent inpatient settings and emphasise the important of providing an environment that actively facilitates compassion for both staff and young people and pays attention to the nature of nested systems in intense ward environments. Staff need to be nourished, valued and compassionately cared for in order to be able to care compassionately for the patients they look after. They need the same elements of compassion as those which they seek to provide. At this current time, when NHS staff are stretched by the Covid-19 pandemic, this study suggests that greater emphasis needs to be placed on providing staff with individual, team and organisational level resources which act to help them to feel compassionately held within the interconnected systems in which they work, in order to be able to continue to provide high level compassionate care for patients.

More research is needed on how changes to the aids and barriers identified in this paper might help address compassion fatigue in this population. In addition, researchers might wish to expand the analysis we present with a consideration of other perspectives on staff compassion, such as those of patients and their families. Our findings also have implications for healthcare workers and nursing management. In particular they highlight that staff need the same elements of compassion that they seek to provide. As such, managers and organisations need to consider interventions that aim to address the six themes outlined in this paper in an effort to improve compassionate care without cost to staff health and wellbeing.

## Supplementary Information

**Additional file 1: Table S1.** Survey results for elements of compassionatecare. **Table S2.** Survey results for aids to compassionate care. **Table S3. **Survey results for barriers to compassionate care.

## Data Availability

The qualitative dataset is not publically available due to possible risk of identifying individual staff members from the transcripts of the focus groups but the quantitative dataset is available on request from the authors upon reasonable request.

## Footnotes

1 Demographic information was not recorded but at a superficial level the sample seemed to be predominantly female and White Caucasian, age ranging from early 20 s to late 50 s. The sample seemed diverse in terms of age and profession but could have been more diverse in other ways.
