# Supplementary material for: “The team needs to feel cared for”: staff perceptions of compassionate care, aids and barriers in adolescent mental health wards
Source: BMC Nurs. 2022 Aug 1;21:206. doi: 10.1186/s12912-022-00994-z (PMC9340707; doi:10.1186/s12912-022-00994-z)
Supplement: Supplementary file 1 — Additional file 1: Table S1. Survey results for elements of compassionatecare. Table S2. Survey results for aids to compassionate care. Table S3. Survey results for barriers to compassionate care. [file 12912_2022_994_MOESM1_ESM.docx]

**Supplementary Tables for Online Publication**

Table S1. Survey results for elements of compassionate care

| **Higher-order theme** | **First-order theme** | **Mean score** | **S.D** |
| --- | --- | --- | --- |
| Emotional Sensitivity | Empathy for the patient | 4.75 | 0.44 |
| Emotional Sensitivity | Helping the patient feel safe | 4.83 | 0.45 |
| Emotional Sensitivity | Sitting with difficult feelings | 4.75 | 0.55 |
| Connection | Emotionally connecting with a patient | 4.64 | 0.59 |
| Connection | Therapeutic touch | 3.00 | 1.31 |
| Valuing | Prioritising patient care | 4.67 | 0.54 |
| Valuing | Having a non-judgemental attitude towards the patient | 4.83 | 0.38 |
| Attending to the whole person | Active Listening | 4.75 | 0.50 |
| Attending to the whole person | Individualising care | 4.61 | 0.60 |
| Attending to the whole person | Spending time with the patient | 4.67 | 0.68 |
| Attending to the whole person | Working as a collective around the person | 4.78 | 0.49 |
| Understanding | Making sense of what is happening | 4.69 | 0.62 |
| Authenticity | Openness | 4.36 | 0.72 |
| Communication | Collaborating with the patient | 4.69 | 0.53 |
| Communication | Explaining what you are doing | 4.67 | 0.59 |
| Communication | Showing the patient that you care | 4.64 | 0.59 |
| Communication | Normalising distress | 4.25 | 0.73 |
| Increasing personal resources | Enabling patient independence | 4.50 | 0.61 |
| Providing practical support | Offering practical help | 4.33 | 0.76 |

Table S2. Survey results for aids to compassionate care

| **Higher-order theme** | **First-order theme** | **Mean** | **S.D.** |
| --- | --- | --- | --- |
| Emotional sensitivity of system | Compassion of other team members | 4.34 | 0.80 |
| Emotional sensitivity of system | Feeling cared for | 4.31 | 0.76 |
| Connection | Team work | 4.63 | 0.49 |
| Connection | Connection with staff team | 4.31 | 0.72 |
| Connection | Connection with patient | 4.09 | 0.92 |
| Being valued | Feeling valued | 4.63 | 0.49 |
| Being valued | Celebrating successes | 4.26 | 0.74 |
| Being attended to as a whole person | Training/Personal Development | 4.37 | 0.69 |
| Understanding | Formulation | 4.77 | 0.43 |
| Understanding | Thinking space | 4.49 | 0.56 |
| Understanding | Individual supervision | 4.60 | 0.55 |
| Authenticity | Authenticity | 4.26 | 1.01 |
| Authenticity | Non-judgement | 4.43 | 0.66 |
| Communication | Respect for different points of view | 4.60 | 0.50 |
| Communication | Team thinking/talking | 4.71 | 0.52 |
| Personal resources | Time away from ward | 4.11 | 0.87 |
| Personal resources | Coping strategies | 4.46 | 0.56 |
| Practical support | Adequate staffing resource | 4.89 | 0.32 |

Table S3. Survey results for barriers to compassionate care

| **Higher order theme** | **First-order theme** | **Mean** | **S.D.** |
| --- | --- | --- | --- |
| Poor emotional sensitivity of system | Constant change | 3.97 | 0.89 |
| Poor emotional sensitivity of system | Prescriptive rules | 3.51 | 0.92 |
| Poor emotional sensitivity of system | Lack of clear boundaries | 4.11 | 0.96 |
| Lack of connection | Lack of connections with team | 3.97 | 0.86 |
| Lack of connection | Impact of patient presentation | 3.57 | 0.92 |
| Low sense of value | Lack of reward | 3.97 | 0.82 |
| Low sense of value | Poor work conditions | 4.20 | 0.68 |
| Lack of attending to the whole staff member | Challenges in personal life | 3.49 | 0.92 |
| Lack of attending to the whole staff member | Impact on home life | 3.57 | 1.01 |
| Lack of understanding | Lack of improvement in patient | 3.51 | 0.82 |
| Lack of authenticity | Self criticism | 3.49 | 0.78 |
| Poor communication | Relationship with management | 4.20 | 0.72 |
| Lack of personal resources | Unhelpful personal coping styles | 3.71 | 1.10 |
| Lack of personal resources | Feeling worn out | 4.29 | 0.86 |
| Lack of practical support | Lack of practical support | 4.06 | 0.77 |
| Lack of practical support | Lack of external resources | 3.91 | 0.92 |
| Lack of practical support | Short staffing | 4.57 | 0.61 |
| Lack of practical support | Physical ward environment | 3.86 | 0.78 |
| Lack of practical support | Lack of time | 4.23 | 0.88 |
| Lack of practical support | Bureaucracy | 4.11 | 0.83 |
